# Clinical Determinants of Myocardial Injury, Detectable and Serial Troponin Levels among Patients with Hypertensive Crisis

**DOI:** 10.7759/cureus.6787

**Published:** 2020-01-27

**Authors:** Giancarlo Acosta, Ahmed Amro, Rodrigo Aguilar, Waiel Abusnina, Niharika Bhardwaj, George Augustine Koromia, Mark Studeny, Affan Irfan

**Affiliations:** 1 Cardiology, Marshall University, Huntington, USA; 2 Internal Medicine, Marshall University, Joan C. Edwards School of Medicine, Huntington, USA; 3 Clinical and Translational Science, Marshall University, Joan C. Edwards School of Medicine, Huntington, USA

**Keywords:** hypertension, hypertensive crisis, troponin, myocardial injury, body mass index, obesity, hypertensive emergency, hypertensive urgency, myocardial infarction

## Abstract

Introduction

There is a high prevalence of hypertensive crisis with myocardial injury, as evidenced by elevation in cardiac troponin levels. The risk factors predisposing patients to developing a myocardial injury, detectable troponin, and increase in serial troponin in this population are not known.

Methods

A retrospective study was designed to include all patients, presenting to the emergency room, diagnosed with hypertensive crisis, using International Classification of Diseases, 10^th^ revision, Clinical Modification (ICD-10-CM) codes between 2016-2018 (n=467). Logistic regression was used to determine the important predictors of myocardial injury evidenced by troponin elevation >99^th^ percentile of upper reference level (URL), detectable troponin (> 0.015 ng/ml), and increase in serial troponin levels.

Results

The 99^th^ percentile of the initial troponin level among all patients was 0.433 ng/ml. A total of 15% had a myocardial injury, and the significant risk factors associated with it were body mass index (BMI) < 30 kg/m2 (odds ratio [OR] 0.50, confidence interval [CI] 0.28-0.89), congestive heart failure (CHF; OR 4.28, CI 2.21-8.25) and prior use of aspirin (OR 1.98, CI 1.08-3.63). About 35% had detectable troponin, and BMI < 30 kg/m^2^ (OR 0.62, CI 0.40-0.97), CHF (OR 3.49, CI 2.06-5.9), elevated creatinine (OR 1.17, CI 1.02-1.34) and age <61 years (OR 0.59, CI 0.38-0.94) were associated with it. The factors associated with an increase in serial troponin were BMI < 30 Kg/m2 (OR 0.56, CI 0.36-0.87), CHF (OR 1.78, CI 1.06-3.0), coronary artery disease (CAD; OR 2.08, CI 1.28-3.36) and non-Caucasian race (OR 0.52, CI 0.29-0.93).

Conclusion

About one-third of patients with the hypertensive crisis have detectable troponin. Still, among these, less than half have troponin levels >99^th^ percentile URL, and the majority of these patients have minimal changes in serial troponin. Low BMI was associated with higher initial and serial troponin levels, and this obesity paradox was stronger among females and older patients.

## Introduction

About one to two percent of patients with hypertension will develop hypertensive crisis in their lifetime [1]. It occurs when systolic blood pressure (SBP) is > 180 and/or diastolic blood pressure (DBP) > 120, and is further classified into hypertensive emergency or urgency depending on the presence of end-organ damage. Hypertensive crisis is a serious condition with a one-year death rate of greater than 70% when left untreated [2]. One commonly affected tissue in extremely elevated blood pressure is the myocardium. Ischemic heart disease is usually precipitated by the increased myocardial oxygen demand, which can occur even in the absence of obstructive coronary artery disease (CAD) or acute atherosclerotic plaque rupture [1, 3, 4]. There is data suggesting that extreme elevations in blood pressure cause endothelial dysfunction, inflammation and pro-thrombotic effects leading to tissue ischemia [5, 6]. This myocardial ischemia can cause angina, electrocardiogram (ECG) changes, and elevation of cardiac biomarkers [1, 7]. 

For the accurate and timely diagnosis of myocardial injury, cardiac troponins (troponin) are the guideline-recommended biomarker. When there is an elevation of troponin of > 99^th^ percentile of the upper reference level (URL), there is myocardial injury [8, 9]. If serial measurements of troponin reveal a variation of > 20% of a previous value in the presence of signs and symptoms of ischemia, then a diagnosis of myocardial infarction (MI) is made [10]. In more recent years, high-sensitivity troponin assays are more commonly being used in clinical practice, and thus improving the detection rates of myocardial injury [9, 11, 12]. However, with increasing minute levels of detected troponin, there are now concerns over the non-CAD specific etiology and possibly identifying adverse cardiovascular phenotype [13, 14]. Therefore, many studies have now suggested that for age, sex and comorbid conditions there should be specific 99^th^ percentile cut-offs for each troponin assay to diagnose myocardial injury [15-19]. 

Despite the high prevalence of hypertensive crisis and possible adverse cardiovascular outcomes among those with elevated troponin, the risk factors associated with myocardial injury, detectable troponin and changes in serial troponin levels are largely not known [7, 20]. The aim of this study was to identify 99^th^ percentile levels for patients admitted with hypertensive crisis, among all patients and different phenotypes, as well as identify the important risk factors associated with troponin levels among these patients. We were particularly interested in the association of troponin levels and body mass index (BMI), as this has never been explored among patients with hypertensive crisis, and because of the known “obesity paradox”. 

## Materials and methods

Methods

A retrospective cohort study was designed based on emergency room visits at Cabell Huntington Hospital / Marshall University Medical Center. Data from the electronic medical record (EMR) was extracted for the time period from October 2016 to October 2018. International Classification of Diseases, 10^th^ revision, Clinical Modification (ICD-10-CM) codes were used for the identification of the qualifying diagnoses. The cohort included all patients ≥ 18 years old with the diagnosis of hypertensive urgency, hypertensive emergency or unspecified hypertensive crisis, by utilizing the following primary diagnostic codes: I16.0, I16.1, and I16.9, respectively. Patients with missing laboratory values (n=43) or those diagnosed with the acute coronary syndrome (n=6) were excluded. 

Variables of Interest

Covariates included baseline demographics: age, gender, race, BMI and smoking status; also comorbidities commonly associated with heart disease were included such as diabetes, hyperlipidemia, cerebrovascular accident, CAD, heart failure, chronic kidney disease (CKD), cerebrovascular accident (CVA) and peripheral vascular disease (PVD). ICD-10-CM codes were used to identify these comorbidities. Other selected variables were: initial creatinine levels, an average of first three systolic and diastolic blood pressures (SBP and DBP), as well as prior outpatient medications including calcium channel blockers, beta-blockers, diuretics, aspirin, angiotensin-converting enzyme inhibitors (ACEI), angiotensin receptor blockers (ARB) and statins. 

Cardiac troponin assay

The troponin assay used during the study period was Siemens Dimension Vista® System (Munich, Germany); limit of detection of 0.015 ng/ml, coefficient of variation (CV) 10% is 0.04 ng/ml and the 99^th^ percentile of URL is ≥ 0.045 ng/ml [21].

Outcomes

The 99^th^ percentile was identified for the whole cohort and by age, gender, BMI, CKD, and race. The primary outcome was myocardial injury (defined as >99^th^ percentile of the URL as per the Fourth Universal Definition of Myocardial Infarction [10]. We also explored the risk factors associated with 1) detectable troponin (>0.015 ng/ml) and 2) changes in serial troponin levels.

Statistical analysis

The data are presented as proportions, means (± standard deviation), and in case of non-normal distribution as median with inter-quartile range (IQR). Comparisons were made using the t-test for normally distributed continuous variables, Mann-Whitney U-test for non-normally distributed continuous variables, Fisher exact test for categorical variables with any field including less than six patients, and chi-square test for the other categorical variables. The Kolmogorov-Smirnov test was used to test for normality. We tested the associations between all covariates and the three outcomes with troponin levels 1) initial >99^th^ percentile of URL; 2) initial detectable troponin; 3) change in serial troponin. Those covariates significantly associated with the outcome in bivariate analyses (with p<0.10) were used to build a full adjusted model. Odds ratio (OR) were calculated with a 95% confidence interval (CI), comparing the significant risk factors associated with the outcome, using binary logistic regression analysis. We also tested for interactions between our main effect variable (BMI) and the subgroups (age, gender and smoking status) using models adjusted for variables similar to those included in the model. A two-sided p-value of ≤0.05 was considered significant for main effects and for interactions.

To test for best fitting model between troponin levels and BMI, scatter plots and curve estimation analyses (tested for linear, logarithmic, inverse, compound, power, S, exponential, growth, logistic) were performed. Quadratic and cubic curves were not included, because scatter plots and the Loess curve did not reveal such an association and were clinically not expected. R^2^ was calculated to assess the performance of the model. Because sensitive cardiac troponins had a non-normal distribution, natural log transformation was performed on values in ng/ml to avoid negative values. Data were analyzed using Statistical Package for Social Sciences (SPSS) software (version 24, IBM Inc, Armonk, USA).

## Results

**Figure 1:**
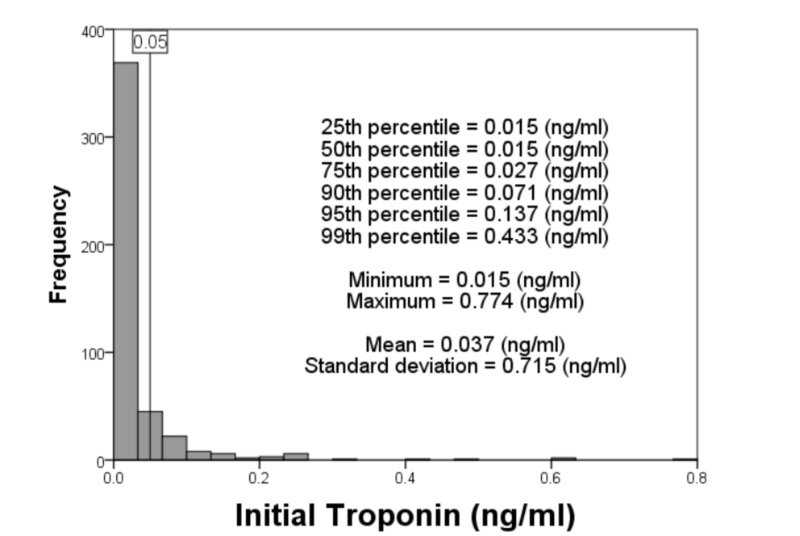
Distribution of initial troponin among all hypertensive crisis patients

A total of 467 patients presenting to the emergency department (ED) with a hypertensive emergency, hypertensive urgency or unspecified hypertensive crisis were included in the study. The median initial troponin value of all patients was ≤ 0.015 ng/ml (i.e., non-detectable), while the 90^th^, 95^th,^ and 99^th^ percentiles were 0.071 ng/ml, 0.137 ng/ml and 0.433 ng/ml respectively (Figure 1 and Table 1). The median, 90^th^, 95^th^ and 99^th^ percentiles of all patients and subgroups are also shown in Table 1. The 99^th^ percentiles were higher among those who were older, female, BMI < 30 kg/m^2^ and CKD. There were not enough African American patients (n=67) to generate 99^th ^percentile; however, the 90^th^ and 95^th^ percentiles were higher in African Americans than among Caucasians. 

**Table 1:** Percentiles of initial troponin among all hypertensive crisis patients and subgroups CKD - chronic kidney disease; BMI - body mass index

	N (%)	50^th^	90^th^	95^th^	99^th^
All patients	467	0.015	0.071	0.137	0.433
Age ≤ 61 year	234 (50%)	0.015	0.089	0.150	0.256
Age > 61 year	233 (50%)	0.015	0.060	0.103	0.612
Male	191 (41%)	0.015	0.090	0.155	0.257
Female	276 (59%)	0.015	0.061	0.112	0.611
BMI ≥ 30 kg/m^2^	229 (49%)	0.015	0.057	0.095	0.295
BMI < 30 kg/m^2^	238 (51%)	0.015	0.090	0.149	0.567
Without CKD	291 (82%)	0.015	0.051	0.088	0.288
CKD	176 (38%)	0.017	0.118	0.186	0.561
Caucasian	396 (85%)	0.015	0.068	0.122	0.500
African American	67 (14%)	0.015	0.104	0.180	-

Myocardial injury (troponin > 99^th^ percentile)

**Figure 2:**
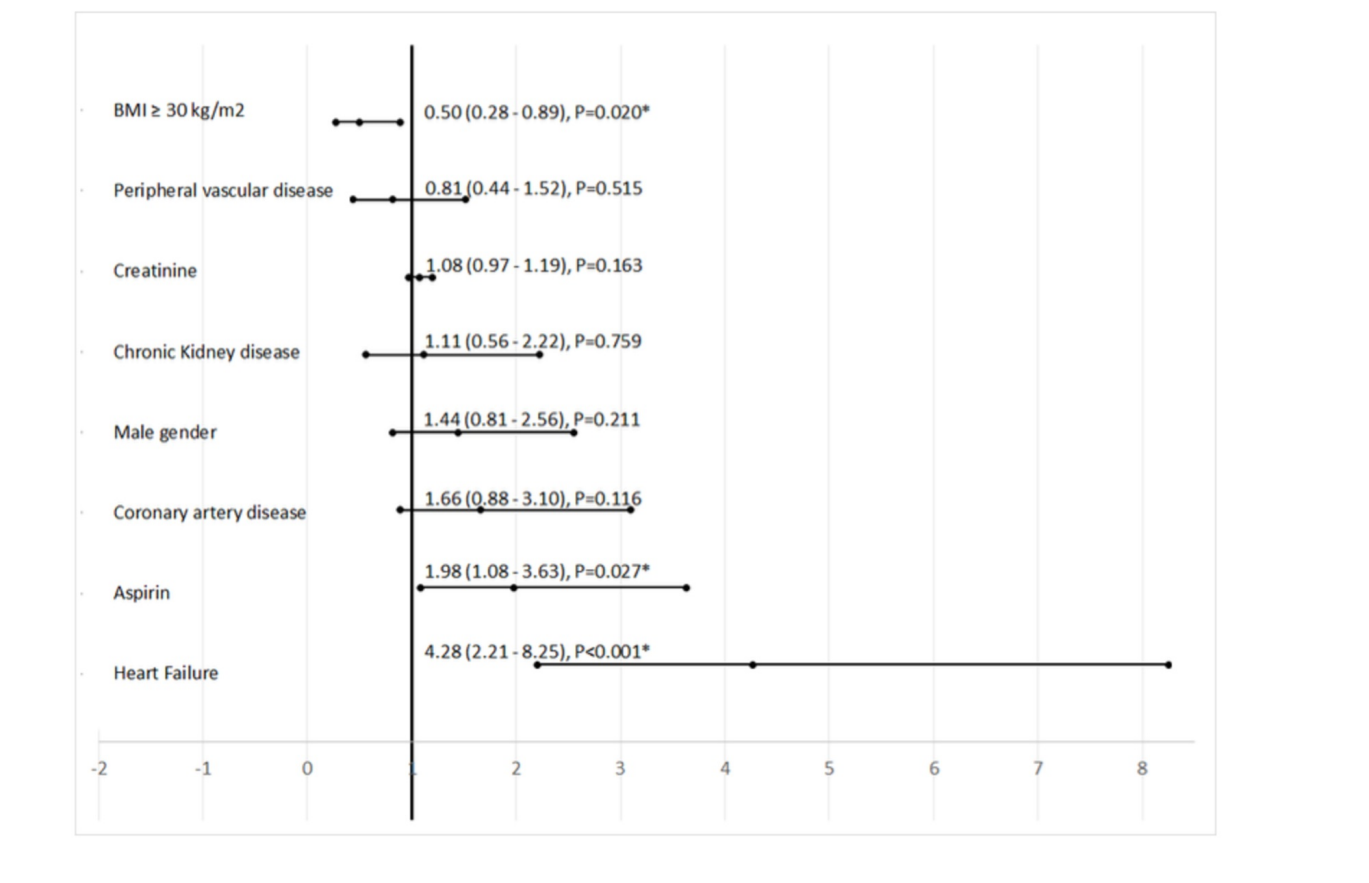
Predictors for myocardial injury from binary logistic regression among those found significant in Table 2 BMI - body mass index

The baseline characteristics and their association with myocardial injury are shown in Table 2. A total of 15% were found to have a myocardial injury. After entering all the covariates that were found significant in Table 2, the binary logistic regressions showed patients with BMI < 30 kg/m^2^, those with a prior diagnosis of heart failure and those with use of aspirin on admission, were significantly associated with higher odds of myocardial injury. Patients with a history of CAD showed a non-significant trend towards myocardial injury (Figure 2). 

**Table 2:** Baseline characteristics and myocardial injury (troponin > 99th percentile) BMI - body mass index; ACEI - angiotensin-converting enzyme inhibitors; ARB - angiotensin receptor blockers

All patients n=467		Troponin > 99^th^ percentile		p-value
		No; n=397 (85)	Yes; n=70 (15)	
At time of emergency department visit				
Age Per year		62 [51 – 71]	59 [52 – 71]	0.309
Age > 61 year		204 (51)	29 (41)	0.124
Male gender		155 (39)	36 (51)	0.052
Caucasian		340 (86)	56 (80)	0.225
BMI Per kg/m^2^		30 [25 – 36]	28 [23 – 34]	0.028
BMI ≥ 30 kg/m^2^		204 (51)	25 (36)	0.016
Mean arterial pressure (mmHg)		138 [127 – 153]	138 [123 – 151]	0.308
Creatinine (mg/dl)		1.06 [0.85 – 1.40]	1.35 [1.01 – 2.26]	<0.001
Prior history				
Diabetes		203 (51)	36 (51)	0.964
Hypertension		382 (96)	69 (99)	0.319
Hyperlipidemia		267 (67)	45 (63)	0.627
Coronary artery disease		166 (42)	45 (64)	<0.001
Cerebrovascular accident		144 (36)	28 (40)	0.551
Chronic kidney disease		135 (34)	41 (59)	<0.001
Peripheral vascular disease		115 (29)	28 (39)	0.107
Heart failure		97 (24)	44 (63)	<0.001
Smoking status	Active smoker	137 (35)	25 (36)	0.776
	Ex-smoker	101 (25)	20 (29)	
	Never-smoker	159 (40)	25 (36)	
Outpatient medications				
Aspirin		79 (20)	23 (33)	0.016
β-blockers		139 (35)	30 (43)	0.208
Diuretics		111 (28)	22 (31)	0.553
ACEI or ARB		147 (37)	25 (36)	0.834
Calcium channel blockers		103 (26)	18 (26)	0.968
Statin		97 (24)	17 (24)	0.979

Detectable troponin (troponin > 0.015 ng/ml)

**Figure 3:**
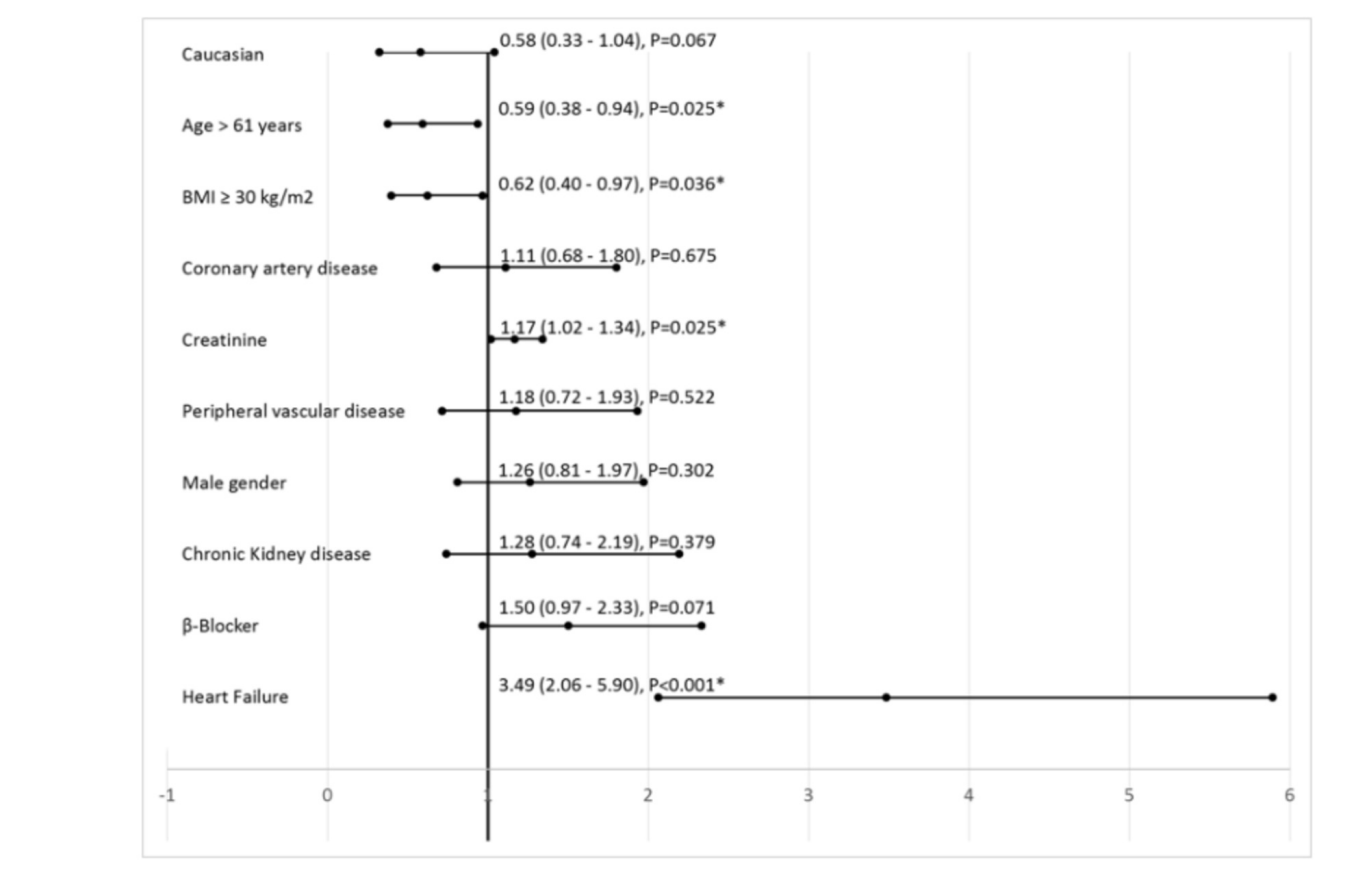
Predictors for detectable troponin from binary logistic regression among those found significant in Table 3 BMI - body mass index

A total of 166 (35%) patients were found to have detectable troponin (Table 3). In the fully adjusted binary regression model, the covariates that were significantly associated with detectable troponin were: BMI < 30 kg/m^2^, age ≤ 61 years, elevated creatinine, and prior diagnosis of heart failure. Non-Caucasians and prior use of beta-blocker showed a non-significant trend towards detectable troponin (Figure 3). 

**Table 3:** Baseline characteristics and detectable troponin (troponin > 0.015 ng/ml) BMI - body mass index; ACEI - angiotensin-converting enzyme inhibitors; ARB - angiotensin receptor blockers

		All patients n=467	Troponin detected		p-value
			No; n=301 (65)	Yes; n=166 (35)	
At time of emergency department visit					
Age per year		61 [51 – 71]	62 [51 – 71]	60 [50 – 70]	0.168
Age > 61 year		233 (50)	161 (54)	72 (43)	0.036
Male gender		276 (59)	112 (37)	79 (48)	0.029
Caucasian		396 (85)	263 (87)	133 (80)	0.037
BMI per kg/m^2^		30 [25 – 36]	30 [25 – 37]	28 [25 – 35]	0.155
BMI ≥ 30 kg/m^2^		229 (49)	159 (53)	70 (42)	0.027
Mean arterial pressure (mmHg)		138 [126 – 153]	137 [126 – 151]	140 [126 – 157]	0.341
Creatinine (mg/dl)		1.06 [0.86 – 1.54]	0.98 [0.81 – 1.22]	1.33 [1.02 – 2.31]	<0.001
Prior history					
Diabetes		239 (51)	151 (50)	88 (53)	0.556
Hypertension		451 (97)	288 (96)	163 (98)	0.153
Hyperlipidemia		312 (67)	202 (67)	110 (66)	0.853
Coronary artery disease		211 (45)	119 (40)	92 (55)	0.001
Cerebrovascular accident		172 (37)	110 (37)	62 (37)	0.863
Chronic kidney disease		176 (38)	85 (28)	91 (55)	<0.001
Peripheral vascular disease		142 (30)	77 (26)	65 (39)	0.002
Heart failure		141 (30)	57 (19)	84 (51)	<0.001
Smoking status	Active smoker	162 (35)	99 (33)	63 (38)	0.407
	Ex-smoker	121 (26)	77 (26)	44 (27)	
	Never-smoker	184 (39)	125 (42)	59 (36)	
Prior outpatient medications					
Aspirin		102 (22)	62 (21)	40 (24)	0.381
β-blockers		169 (36)	96 (32)	73 (44)	0.009
Diuretics		133 (29)	86 (29)	47 (28)	0.953
ACEI or ARB		172 (37)	110 (37)	62 (37)	0.863
Calcium channel blockers		121 (26)	72 (24)	49 (30)	0.186
Statin		114 (24)	75 (25)	39 (24)	0.732

BMI and troponin levels

**Figure 4:**
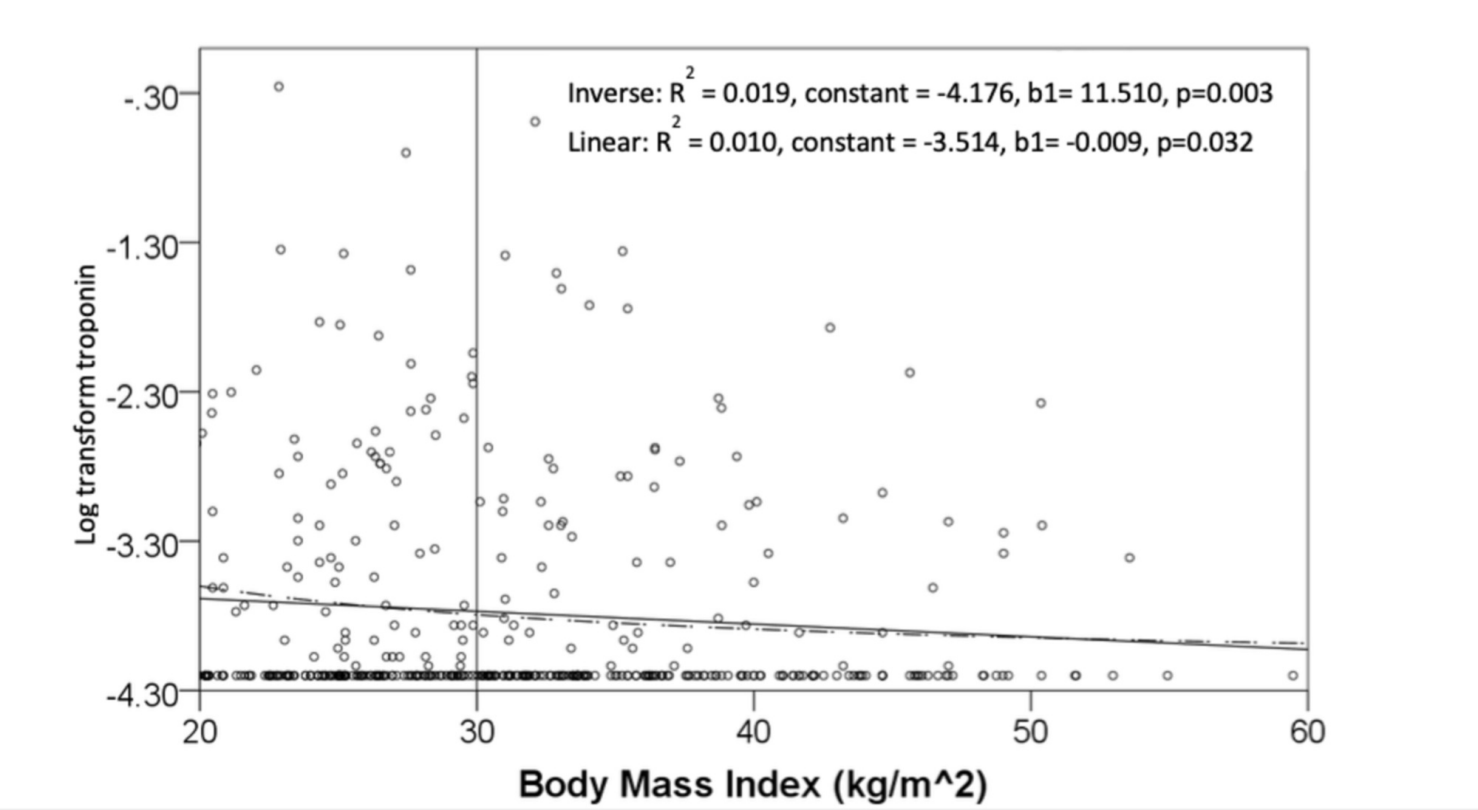
Relationship of initial log transform troponin levels and body mass index in kg/m2

About half of our cohort were defined as obese (BMI ≥ 30 kg/m2). In obese patients (n=229, 49%), the 95^th^ and 99^th^ percentile of the initial troponin values were 0.095 ng/ml and 0.295 ng/ml, respectively, whereas that among non-obese patients were 0.149 ng/ml and 0.567 ng/ml, respectively. Lower BMI had a significantly higher association with myocardial injury, detectable troponin, and positive change in serial troponin. It also had a significant negative linear relationship with initial troponin levels. Among different models applied to the association between log-transformed high-sensitivity cardiac troponin and BMI, inverse function achieved the highest significant R^2^ (R^2^ = 0.019, constant = -4.176, b1= 11.510, p=0.003) (Figure 4). 

Serial troponin levels

**Figure 5:**
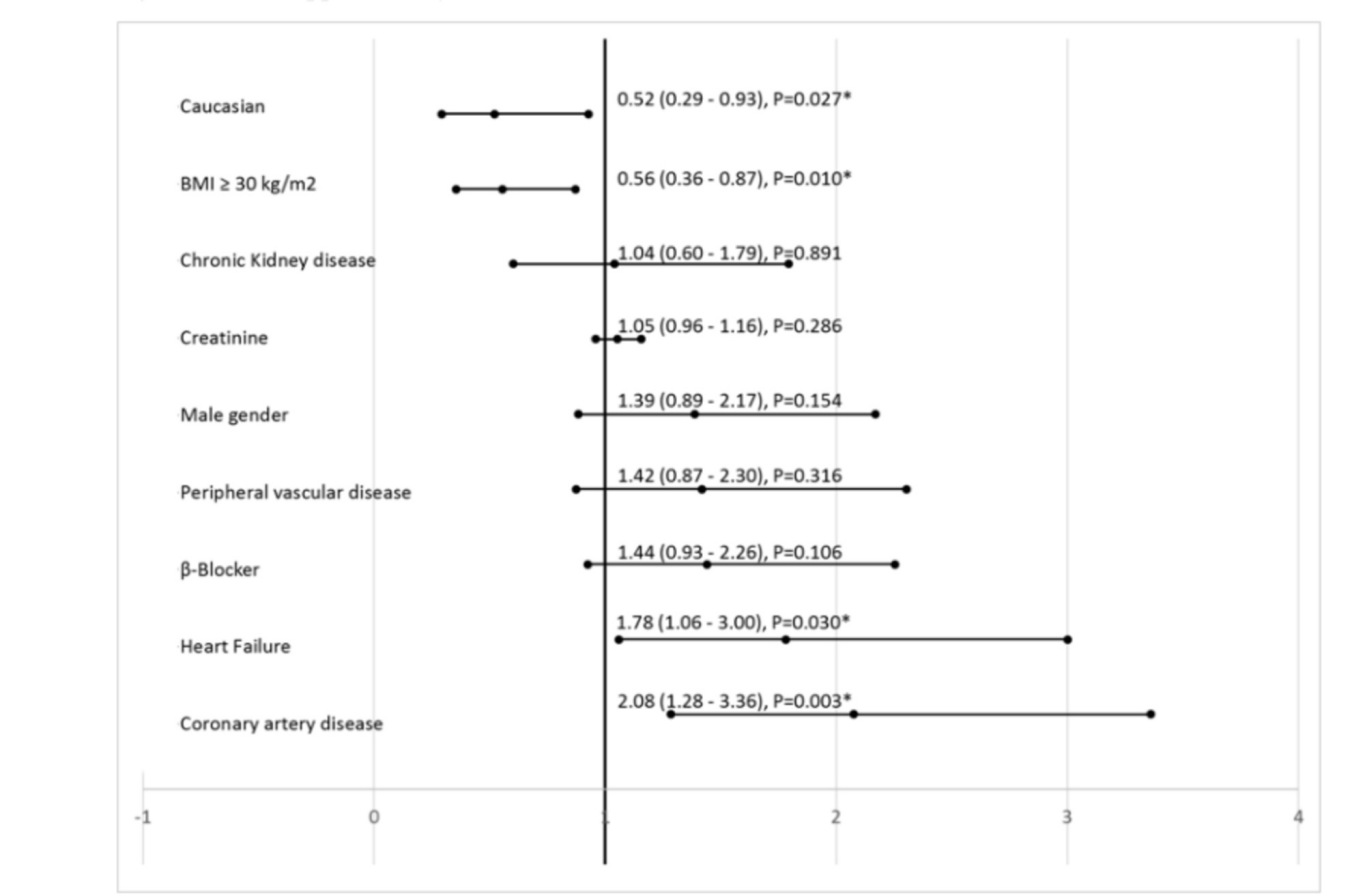
Predictors for positive serial troponin from binary logistic regression BMI - body mass index

Among all patients, 135 (29%) had a positive change in serial troponin levels compared to their initial troponin (Table 4). Patients with BMI < 30 kg/m^2^, non-Caucasians, prior diagnosis of heart failure, and prior CAD were significantly associated with an increase in troponin levels from baseline. Prior use of beta-blocker showed a non-significant trend towards change in troponin (Figure 5). The majority of patients who did not have a change in serial troponin (n=332, 71%), also did not have initial troponin > 99^th^ percentile (95%) (Table 5). Only four patients (0.9% of the whole cohort) had a positive change in relative troponin in the range of ≥20% from the baseline troponin levels. 

**Table 4:** The proportion of patients with and without an increase in serial troponin levels by the initial troponin detected and >99th percentile

Initial troponin	Serial troponin positive		p-value
	No; n=332 (71)	Yes; n=135 (29)	
Not detected	263 (79)	0 (0)	< 0.001
Detected	69 (21)	135 (100)	
≤ 99^th^ percentile	315 (95)	82 (61)	< 0.001
> 99^th^ percentile	17 (5)	53 (39)	

**Table 5:** The proportion of patients with and without an increase in serial troponin levels by the initial troponin detected and >99th percentile

Initial troponin	Serial troponin positive		p-value
	No; n=332 (71)	Yes; n=135 (29)	
Not detected	263 (79)	0 (0)	< 0.001
Detected	69 (21)	135 (100)	
≤ 99^th^ percentile	315 (95)	82 (61)	< 0.001
> 99^th^ percentile	17 (5)	53 (39)	

There was also evidence of effect modification by age and by gender, where the association between low BMI and myocardial injury (initial troponin >99^th^ percentile) was stronger in older age group (interaction p-value = 0.003) and females (interaction p-value = 0.017). There was no effect modification by the subgroups between the association of BMI and detectable troponin and positive serial troponin levels (Table 6).

**Table 6:** Association between body mass index and myocardial injury, detectable troponin and serial troponin changes in subgroups

	Myocardial injury		Detectable troponin		Positive serial troponin levels	
	Odds ratio (95% CI)	Interaction p-value	Odds ratio (95% CI)	Interaction p-value	Odds ratio (95% CI)	Interaction p-value
Age ≤ 61 years	0.98 (0.44, 2.15)	0.003	0.96 (0.48, 1.91)	0.315	0.78 (0.39, 1.55)	0.981
Age > 61 years	0.13 (0.04, 0.39)		0.49 (0.26, 0.93)		0.44 (0.23, 0.84)	
Female	0.23 (0.09, 0.56)	0.017	1.09 (0.53, 2.25)	0.158	0.62 (0.29, 1.30)	0.843
Male	1.01 (0.45, 2.29)		0.42 (0.23, 0.76)		0.50 (0.28, 0.91)	
Active smoker	0.61 (0.19, 1.98)	0.792	0.50 (0.22, 1.15)	0.472	0.66 (0.28, 1.54)	0.421
Ex-smoker	0.27 (0.07, 1.04)		1.10 (0.46, 2.62)		0.42 (0.15, 1.21)	
Never-smoker	0.40 (0.15, 1.10)		0.44 (0.34, 1.53)		0.67 (0.32, 1.38)	

## Discussion

This is the first study to establish 99^th^ percentiles of troponin levels among patients with hypertensive crises and subgroups (age, gender, BMI, CKD, and race). We identified the significant risk factors that predict myocardial injury, detectable troponin, and increase in serial troponin levels among hypertensive crisis patients. There are several major findings and important clinical implications of the study. 

First, in our cohort, 15% had troponin >99^th^ percentile of URL and the 99^th^ percentile of troponin for all patients was 0.433 ng/ml (almost 10 times higher of that recommended by the manufacturer to identify myocardial injury [0.045 ng/ml]). However, only < 1% had a relative change in serial troponin levels of greater than 20%, suggesting the majority of hypertensive crisis patients will be categorized as chronic myocardial injury. This provides evidence that serial troponin levels can potentially, more reliably, differentiate hypertensive crisis from ACS patients, rather than one-time troponin level. The second major finding is that patients with lower BMI, prior CHF and prior use of aspirin were the only factors significantly associated with myocardial injury. Interestingly, we also found the negative association between BMI and the myocardial injury was significantly stronger among female and older patient groups. Third, low BMI and prior diagnoses of CHF were the only variables consistently associated with all outcomes (myocardial injury, detectable troponin, and increase in serial troponin levels). 

Other minor findings are 1) there was no association with presenting blood pressure (SBP, DBP, mean arterial blood pressure, pulse pressure) and troponin levels among all patients and in subgroups (data not shown); 2) elevated creatinine contributes to troponin being detectable but not with myocardial injury; 3) younger compared to older age group patients had higher association with detectable troponin (not with myocardial injury), and overall troponin levels had a significant negative linear relationship with age; 4) no significant difference between troponin levels and gender after adjusting to significant covariates; 5) non-Caucasians were more likely to have detectable troponin and increase in serial troponin, but not myocardial injury.

Body Mass Index

In our study, the 99^th^ percentile of the troponin level for patients with obesity was significantly lower than non-obese patients. Patients with BMI < 30 kg/m2 were significantly associated with higher odds of myocardial injury, detectable troponin, and an increase in serial troponin levels. After adjusting for significant covariates, there was a negative linear and inverse relationship between BMI and troponin level. Although it is well known that obesity is an independent risk factor for developing heart disease, there are several large databases that have demonstrated that obese patients have a better short and long term prognosis compared to normal/low weight, among those with CAD and heart failure patients; commonly referred to as the “obesity paradox” [22-28]. We for the first time also found a similar obesity paradox phenomenon among hypertensive crisis patients. The mechanisms by which normal BMI patients have a higher prevalence of detectable and elevated troponin is not clear. Smoking is known to have a strong independent inverse association with adiposity and a possible explanation for the paradox [29]. However, we did not find any interaction effect of smoking status on the association between BMI and troponin levels. Other possible mechanisms to this phenomenon could be that despite obesity being characterized by increased circulating blood volume, systemic vascular resistance and left ventricular mass (which contribute to development of hypertension) obese patients have 1) lower baseline levels of the renin-angiotensin system and attenuated neurohormonal responses to stress; 2) increased nutritional and metabolic reserves; 3) might better tolerate cardio-protective medications because of elevated blood pressures [28, 30]. Lastly, this obesity paradox could be erroneous due to the possible higher undiagnosed systemic conditions or more severe illnesses and unintentional weight loss among lean patients. Interestingly, we found that the obesity paradox to be stronger among an older group and female patients, a finding which is supported by another study which showed obesity was associated with lower in-hospital mortality in patients ≥70 years with myocardial infarction, with a more pronounced paradox in women. Similar female gender-specific and older age-specific obesity paradox has been found in other studies [28].

Other clinical variables and implications

Even though the 99^th ^percentile was lower among patients <61 years, lower creatinine and CKD, after adjusting for significant covariates, patients <61 years and elevated creatinine were independently associated with detectable troponin, but not with myocardial injury and serial troponin levels. There was a strong significant negative linear relationship between initial troponin and age and creatinine. This is in contrast to previous studies that have suggested a higher URL for older patients group. The mechanism for the negative relationship between troponin and age among these patients is not known. A possible explanation could be that secondary hypertension is more common among the younger patient group, and manifest different phenotype, etiology, and mechanisms of troponin leak compared to older counterparts. Another possible explanation for such a finding could be that younger patients tend to be less compliant with their medications leading to more extreme elevations in blood pressure and thus causing more myocardial injury. There is also less awareness, treatment, and control of hypertension in younger adults, and this adverse outcome is possibly reflected by higher troponin levels among our study patients.

In our study, apart from BMI, those patients with a prior diagnosis of CHF, was the only other covariate, that had significantly higher odds of having a myocardial injury, a detectable troponin, and serial troponin levels. This is consistent with prior studies showing that most patients with stable chronic heart failure have detectable troponin, even when the heart failure is chronic and the patient is euvolemic. Patients with prior CAD had significantly higher odds of having detectable troponin levels and a non-significant trend towards myocardial injury, as they are more likely to have a myocardial injury because of increased myocardial oxygen demand and limited supply [10]. 

Limitations

Our study was single-center, retrospective and our data set was limited to the use of ICD-10-CM data (from October 2015). Based on our data, we cannot investigate the different causes of troponin elevation. We could not identify other acute conditions (such as hypoxia, pulmonary embolism, anemia, etc), that are known to cause troponin elevation and may coexist at the time of admission. Despite this is the first study, we were limited by sample size. There are several other clinical tools (such as history, electrocardiogram, echocardiogram, etc) and methods of obesity measurement (such as body surface area, waist circumference, lean body mass, etc) which were unavailable and could potentially better explain troponin levels and warrants further research. 

## Conclusions

About one-third of patients with the hypertensive crisis have detectable troponin. Still, among these patients, less than half have troponin levels consistent with myocardial injury, and the majority of these patients have minimal changes in serial troponin. Low BMI, prior CHF, and use of aspirin are independently associated with myocardial injury among these patients. Low BMI is significantly associated with higher initial and serial troponins. The significant negative association between BMI and the myocardial injury was stronger among female and older patient groups. These observations enhance our knowledge of pathophysiology, risk factors, and the clinical importance associated with baseline and serial troponin levels among hypertensive crisis patients.
